# GSTA3 Attenuates Renal Interstitial Fibrosis by Inhibiting TGF-Beta-Induced Tubular Epithelial-Mesenchymal Transition and Fibronectin Expression

**DOI:** 10.1371/journal.pone.0160855

**Published:** 2016-09-07

**Authors:** Yun Xiao, Jishi Liu, Yu Peng, Xuan Xiong, Ling Huang, Huixiang Yang, Jian Zhang, Lijian Tao

**Affiliations:** 1 Division of Nephrology, Xiangya Hospital, Central South University, Changsha, China, 410078; 2 Division of Nephrology, The First Affiliated Hospital of Guangzhou Medical University, Guangzhou, China, 510120; 3 State Key Laboratory of Medical Genetics of China, Central South University, Changsha, China, 410078; 4 Division of Digestive Medicine, Xiangya Hospital, Central South University, Changsha, China, 410078; 5 Department of Microbial Infection and Immunity, The Ohio State University, Columbus, Ohio, United States of America; Universita degli Studi di Torino, ITALY

## Abstract

Tubular epithelial-mesenchymal transition (EMT) has been widely accepted as the underlying mechanisms of renal interstitial fibrosis (RIF). The production of reactive oxygen species (ROS) plays a vital role in tubular EMT process. The purpose of this study was to investigate the involved molecular mechanisms in TGF-beta-induced EMT and identify the potential role of glutathione S-transferase alpha 3 (GSTA3) in this process. The iTRAQ screening was performed to identify protein alterations of the rats underwent unilateral-ureteral obstruction (UUO). Protein expression of GSTA3 in patients with obstructive nephropathy and UUO rats was detected by immunohistochemistry. Protein and mRNA expression of GSTA3 in UUO rats and NRK-52E cells were determined by Western blot and RT-PCR. siRNA and overexpression plasmid were transfected specifically to assess the role of GSTA3 in RIF. The generation of ROS was measured by dichlorofluorescein fluorescence analysis. GSTA3 protein and mRNA expression was significantly reduced in UUO rats. Immunohistochemical analysis revealed that GSTA3 expression was reduced in renal cortex in UUO rats and patients with obstructive nephropathy. Treating with TGF-β1 down-regulated GSTA3 expression in NRK-52E cells, which have been found to be correlated with the decreased expression in E-cadherin and megalin and increased expression in α-smooth muscle actin. Furthermore, knocking down GSTA3 in NRK-52 cells led to increased production of ROS and tubular EMT, whereas overexpressing GSTA3 ameliorated ROS production and prevented the occurrence of tubular EMT. GSTA3 plays a protective role against tubular EMT in renal fibrosis, suggesting GSTA3 is a potential therapeutic target for RIF.

## Introduction

Progressive renal interstitial fibrosis (RIF) is the final common pathologic change for a number of independent and overlapping cellular and molecular pathways in tubulointerstitial injury of chronic kidney diseases (CKD) [1]. The extent of tubulointerstitial injury correlates closely with long-term renal function and is an important predictor of renal impairments. However, how to prevent CKD remains elusive. Understanding the mechanisms of RIF is essential in establishing novel interventional strategies to halt or even reverse renal fibrosis. Renal tubular epithelial to mesenchymal transition (EMT) has been recognized as the fact of loss of epithelial cell phenotype and a concomitant development of mesenchymal phenotype [2]. Therefore, tubular EMT is believed to be the core part of RIF [3] Phenotypically, tubular EMT are associated with down-regulation of expression of intercellular epithelial adhesion molecules such as E-cadherin and up-regulation of the robust markers of mesenchymal cells such as α-smooth muscle actin (α-SMA) and vimentin [4,5]. It is believed that tubular EMT is responsible for the dissociation of renal tubular epithelial cells, tubular atrophy, accumulation in the interstitium of fibroblasts with the phenotypic appearance of myofibroblasts, and secretion of large amounts of extra cellular matrix (ECM) [6].

A number of factors have been shown to affect the occurrence of renal tubular EMT including oxidative stress, inflammation, fibroblasts proliferation and activation, and apoptosis [7,8]. Among these factors, oxidative stress in the pathogenesis of RIF has grown in scope and understanding in recent years [9]. Mice deficient for endogenous antioxidant enzyme CAT are more susceptible to unilateral ureteral obstruction (UUO)-induced renal damage than normal wild type (WT) mice. Furthermore, increased renal concentrations of ROS have been observed in obstructed kidneys, together with decreased activities of the major protective antioxidant enzymes SOD, CAT, and glutathione peroxidase [10]. It has been shown that progression of RIF correlates with increased activity of ROS, augmented expression of collagen deposition and α-SMA, and loss of E-cadherin and megalin in renal tissue in animals undergoing UUO, suggesting that oxidative stress plays an important role in promoting the tubular EMT [11]. However, the mechanisms underlying this process are still unclear.

The intracellular signaling pathways leading to initiation of EMT remain largely unknown. In the present study, we have used isobaric tags for relative and absolute quantitation (ITRAQ), a new tool for quantitative mass spectrometry, to analyze the differential expression of proteins in the kidneys of sham and UUO rats. Glutathione S-transferase alpha-3 (GSTA3), a member of an important family of detoxifying and cytoprotective enzymes [12] was identified to be the most significantly down-regulated protein in the renal cortex of UUO rats. GSTA3 plays a critical role in various diseases associated with oxidation-regulating proteins [13]. However, whether GSTA3 plays a role in the development of RIF is currently unknown. In this study, we would examine the molecular and cellular basis of RIF and report the role of GSTA3 in the progression of tubulointerstitial fibrosis.

## Materials and Methods

This study was approved by the ethical committee of Central South University.

### Antibodies and reagents

NRK-52E cells were purchased from American Type Culture Collection (Rockville, Md., USA). Dulbecco’s modified Eagle’s medium (DMEM), fetal bovine serum (FBS), penicillin/streptomycin, and Carboxy-DCFDA were obtained from Invitrogen (Carlsbad, Calif., USA). SB431542 and antibodies against α-SMA and β-tubulin were purchased from Sigma-Aldrich (St. Louis, MO, USA). The antibody against E-cadherin was purchased from BD Biosciences (San Jose, CA, USA). Antibodies against GSTA3 and Fibronectin (FN) were obtained from Abcam (Cambridge, UK). Horseradish-peroxideconjugated secondary antibodies for Western blot were from the Jackson ImmunoResearch Laboratories (West Grove, PA, USA), and secondary antibodies for immunohistochemistry were from GBI (Mukilteo, WA, USA). The enhanced chemiluminescence kit for Western blotting was from GE Healthcare (Buckinghamshire, UK). Recombinant human TGF-β1 was purchased from Peprotech (Rocky Hill, NJ, USA). iTRAQ™ Reagents were purchased from Applied Biosystems (USA). DCFH-DA was purchased from Invitrogen (Carlsbad, CA, USA)

### Animal model

Male Sprague-Dawley rats (180-220g, female, 6–8 weeks old, n = 10) were obtained from Slac Laboratory Animal (Shanghai, China). They were given free access to commercial standard rat chow (Hunan Shi Lai Ke Jing Da Experimental Animal Co. Ltd., China) and tap water and housed in stable conditions at 22°C with a 12 h light/dark cycle during the entire study unless mentioned somewhere else. Sprague-Dawley rats were divided into 2 groups (5/group): Sham operation (SHM) and UUO. Under anesthesia with 10% chloral hydrate (0.4 ml/100 g), UUO was induced by ligation of the left ureter according to the procedure described previously [14]. Heart rate was monitored to ensure adequate levels of anesthesia. Sham operations were performed without ligation. Immediately post-surgery, animals were administrated buprenorphine (0.03 mg/kg, s.c.) for post-operative pain. Animals were monitored twice/day, and weighed daily. All rats were sacrificed at day 14. Left ventricular blood was collected and tested for serum creatinine and uric acid. Half of the left kidneys were fixed in 10% neutral-buffered formalin for H&E and Masson’s trichrome staining or immunohistochemistry, and the remaining half was frozen in liquid nitrogen for Western blot or RT-PCR. All animal studies were approved by the Institutional Animal Care and Use Committee of Xiangya School of Medicine, Central South University.

### Quantitative proteomics using iTRAQ labeling and mass spectrometry

For iTRAQ screening, renal cortex isolated from SHM rats and UUO rats were lysed several times by freeze-thawing, followed by sonication. The extracted proteins were reduced, alkylated as described in the iTRAQ protocol (Applied Biosystems, Foster City, CA, USA). Isobaric tagging iTRAQ reagent (1 unit in ethanol) was added directly to the protein digest (70% ethanol final) and the mixture was incubated. Proteins were subjected to the conventional procedure with off-line 2D LC-MS/MS. Peptides were eluted with a linear gradient. Then the LC MS/MS analysis was conducted with samples injected into the trap column. To identify the peptides, database search were performed with ProteinPilot 2.0.1 software (Applied Biosystems) using Paragon and ProGroup algorithms against the Swiss-Prot rat database [15]. We selected differentially expressed proteins using peptide confidence >95% criteria, and fold-changes>1.2 or <0.8 were set as cut-off values.

### Histopathological examination

Tissues were fixed in 10% neutral-buffered formalin, dehydrated in graded alcohol, and embedded in paraffin. Paraffin sections (3–5μm thick) were stained with HE. The tubulointerstitial damage index was graded as described previously [16]. To further analyze the degree of interstitial collagen deposition, sections were stained with Masson’s-trichrome as mentioned before [17].

### Patients and renal histology

The obstructive nephropathy renal tissues were obtained from eight patients with kidney stones that underwent a lithotomy at Department of Urology, Xiangya Hospital, Central South University, China. Negative control tissue was removed from the unaffected poles of eight kidneys with renal cell carcinoma. All cases were diagnosed by two individual pathologists in a double-blind manner. The written informed consent was obtained from all the subjects prior to the study. The use of human subjects was approved by the Medical Ethics Committee (MEC) of Central South University. Research content and methods complied with the specifications and requirements of the MEC.

### Immunohistochemistry

Immunohistochemical method was used to determine the expression of GSTA3 in human and rat kidneys. The immunohistochemistry of GSTA3 was performed in paraffin sections using a microwave-based antigen retrieval technique. After immunostaining, the sections were counterstained with H&E. Interstitial staining of GSTA3 was measured by a blinded observer using computerized morphometry (Leica QWin 2.8 software) as described before [18].

### Cell culture and treatment

NRK-52E cells were maintained in DMEM supplemented with 8% FBS, 100 U/ml penicillin and 100μg/ml streptomycin. The NRK-52E cells were seeded in 6-well plates with approximately 60–70% confluence for 24 h, and then changed to serum-free medium and incubated for 24 h before TGF-β1 treatment. Recombinant human TGF-β1 was added to the culture at a final concentration of 10 ng/ml (determined by a preliminary experiment). For detection of GSTA3, FN, α-SMA and E-cadherin, cells were pretreated with SB431542 (TGF-β superfamily type I activin receptor-like kinase inhibitor) (10ng/ml) for 1 h. The cells were treated for 48h before protein isolation. Each experiment was replicated 3 times.

### Transient transfection and gene silencing

NRK-52E cells were transiently transfected with pEX2-GSTA3 or pEX2 using Lipofectamine 2000 according to the manufacturer's instructions. For GSTA3 knockdown, silencer select pre-designed siRNA for GSTA3 (sense 5’-GATCCGCCAGTCCTTCACTACTTCT AGTGCTCCTGGTTGGAAGTAGTGAAGGACTGGCTTTTTTAT-3’, antisense 5’-CGATAAAAAAGCCAGTCCTTCACTACTTCCAACCAGGAGCACTAGAAGTAGTGAAGGACTGGCG-3’) was used. Silencer negative control siRNA without mammalian homology was used as negative control (control siRNA). Cell cultures of 60–70% confluence were prepared in 6-well plates and siRNA was introduced using Lipofectamine 2000 according to the manufacturer's instructions. In brief, 4 μg siRNA (10 μM solution) and 10 μL Lipofectamine 2000 were diluted separately with 250 μL Opti-MEM I Reduced Serum Medium and kept at room temperature for 5 min. The diluted siRNA and Lipofectamine 2000 were mixed gently followed by incubation for 30 min at room temperature. 500 μL of siRNA-Lipofectamine complex was added to each well containing 1500 μL DMEM without antibiotics. After incubation for 6 h, the medium was replaced with normal DMEM, and the cells were maintained for an additional 18h. Cells were treated with TGF-β1 and harvested for protein extraction after 48 h. Overexpression and knockdown of GSTA3 were determined by Western blot analysis.

### Gene expression of GSTA3, α-SMA, E-cadherin and FN

Total RNA was isolated from NRK-52E cells and frozen renal tissues using TRIzol Reagent (Invitrogen) according to the manufacturer’s instructions. Equal amounts of RNA (2 μg) were reverse-transcripted into cDNA using the Transcriptor First Strand cDNA Synthesis Kit (Roche; IN, USA). The specific primers for GSTA3, α-SMA, E-cadherin, Fibronectin, Collagen I and β-actin were designed from their GenBank sequences, synthesized by Bio Basic (Shanghai, China), and are listed in Table 1. The RT-PCR quantitation for individual target mRNA expression was performed on an ABI Model 7500 Sequence Detector (Applied Biosystems, Foster City, CA, USA) using a TaKaRa real-time PCR kit. The amplified PCR products were quantified by measuring the target and β-actin mRNA calculated cycle thresholds (CT). The amount of specific mRNA in each sample was calculated from the standard curve and normalized with β-actin [19].

**Table 1:** Nucleotide sequences of the primers used for real-time polymerase chain reaction.

Genes	forward primer(5'-3')	reverse primer(5'-3')
GSTA3	AACCGTTACTTTCCTGCCTTTG	GCCCTGCTCAGCCTATTGC
α-SMA	CTAAGGCCAACCGGGAGAAA	CCAGAGTCCAGCACAATACCA
E-cadherin	TGTTGATAGCGTGCCCTTTG	GTTCCGATTGCTTGCCTTTT
Fibronectin	GTGTCCTCCTTCCATCTTC	CAGACTGTCGGTACTCACG
Collagen I	TCAGGGGCGAAGGCAACAGT	TTGGGATGGAGGGAGTTTACACGA
β-actin	CGTTGACATCCGTAAAGACC	GGAGCCAGGGCAGTAATCT

### Protein extraction and Western Blot analysis

Total proteins from kidneys or NRK-52E cells were collected as described previously [20]. The cell lysate containing 20 μg total protein was separated on 10% sodium dodecyl sulfate-polyacrylamide gel under the reducing condition, and transferred onto polyvinylidene difluoride membranes (Millipore; Billerica, MA, USA). Membranes were incubated overnight at 4°C with primary antibodies against α-SMA (1:3000; Sigma), E-cadherin (1:3000; BD biosciences), GSTA3 (1:500; Abcam, Cambridge, UK), FN (1:5000; Abcam, Cambridge, UK), megalin (1:1000; Santa Cruz), p-Smad2/3 (1:1000; Cell signaling) and followed by horseradish-peroxide-conjugated secondary antibodies. Bands were visualized by enhanced chemiluminescence and quantified using Glyko Bandscan 5.0 (Glyko, Novato, CA, USA). Results were expressed as the percentage change in the mean band density as compared with the control values.

### Detection of ROS and SOD activity

The changes of intracellular ROS levels were determined by measuring the oxidative conversion of cell-permeable 2’,7’-dichlorofluorescein diacetate (DCFH-DA) to fluorescent dichlorofluorescein (DCF) by flow cytometry. In brief, the treated cells were washed with PBS solution and incubated with DCFH-DA (5mM, Invitrogen, USA) at 37°C for 20 min. The cells were re-suspended in 1 ml of PBS. DCF fluorescence was then measured by flow cytometry. Fluorescence data were expressed as percentage change to untreated samples. The activity of SOD was determined using kit from Nanjing Jiancheng Biological Company (Nanjing, China).

### Statistical analysis

Results were expressed as means±SEM. Statistical tests were performed using SPSS 16.0 Software (SPSS16.0, Inc., Chicago, Ill, USA). Unpaired Student's t test was used to compare the means of two groups. One-way ANOVA was applied to compare the means of three or more groups. *P*<0.05 was considered to be significant.

## Results

### Kidneys from UUO rats developed RIF and displayed significantly tubular EMT changes

**Fig 1:**
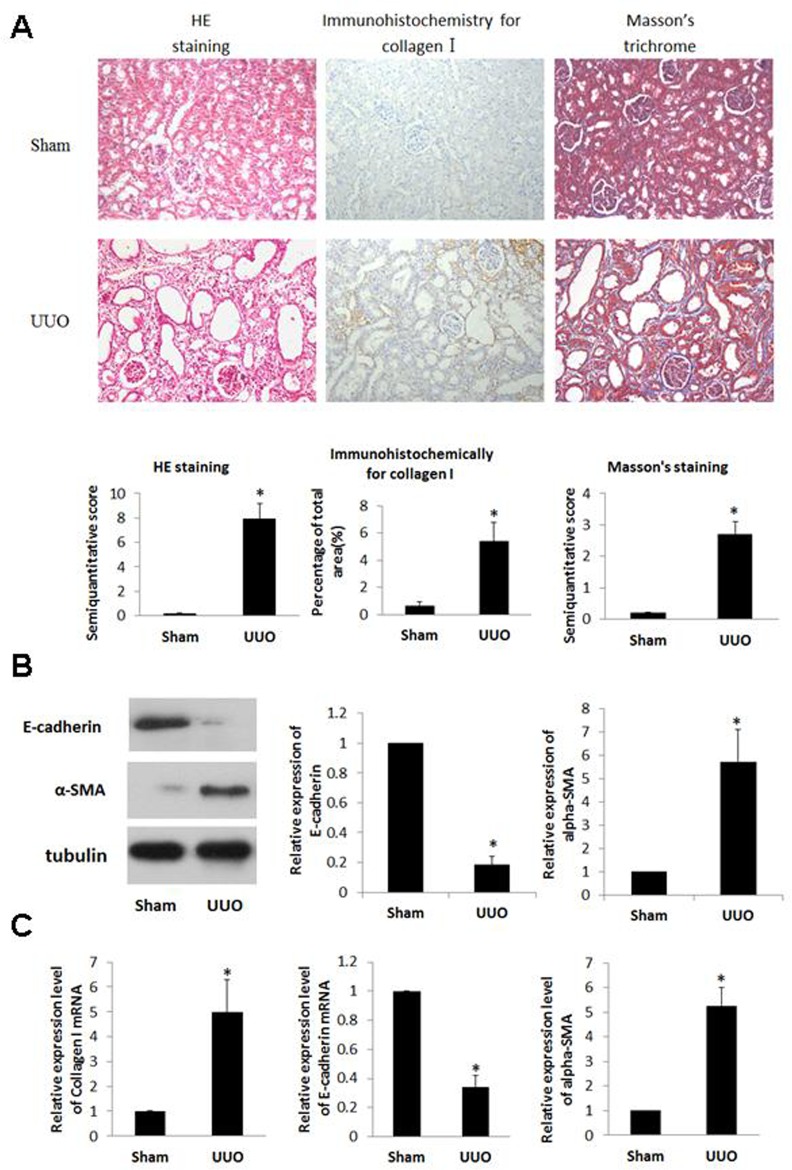
**A) Renals of UUO rats underwent display significant pathological changes and an increase in collagen I expression**. Sprague-Dawley rats were divided into 2 groups (5/group): Sham- operation (SHM) and UUO. All rats were sacrificed at day 14. renal cortex stained with HE, Masson’s trichrome or immunohistochemical for collagen I (×200). Semiquantitative analysis of tubulointerstitial damages as the degree of interstitial collagen deposition and collagen I expression in the renal cortex of rats from each group. Data expressed as means±S.E. *p<0.05 vs. SHM. **B) Expression of α-SMA protein was increased and that of E-cadherin protein was decreased in renal cortex of UUO rats.** The renal cortexes from different rats were lysed. The cell lysates were blotted with anti-α-SMA and anti-E-cadherin antibody. **C) Expression of α-SMA and Collagen I mRNA increased and that of E-cadherin mRNA decreased in renal cortex of UUO rats.** The mRNA from different rats was extracted, and the mRNA for α-SMA, E-cadherin, and Collagen I were detected by real-time PCR. Data expressed as means±S.E. *p<0.05 vs. SHM.

Tubulointerstitial injury including tubular dilatation, atrophy, vacuolization, and interstitial fibrosis were observed in UUO kidneys. Masson’s trichrome staining was used to determine total collagen expression in the interstitial space. An increase of collagen levels was observed in the cortex of UUO rats (*p*<0.05). Consistent with this result, collagen I staining was significantly increased in the interstitium of UUO kidneys as revealed by immunohistochemistry staining (*p*<0.05) (Fig 1A).

E-cadherin and α-SMA are the key biomarkers for tubular EMT responses [21]. Up-regulated expression of α-SMA and down-regulated expression of E-cadherin were observed in previous human and pre-clinical renal fibrosis models [22]. In present study, we have found that UUO induced increases in the protein and mRNA expression of α-SMA, whereas the protein and mRNA expression of E-cadherin was decreased compared to the control (Fig 1B). Collagen I is a major ECM component accumulated in the process of renal tubulointerstitial fibrosis. Compared to SHM, UUO rats exhibited a 5-fold increase in collagen I mRNA expression in the kidney (Fig 1C).

### GSTA3 expression was significantly decreased in renal cortex of UUO rats and patients with obstructive nephropathy

**Fig 2:**
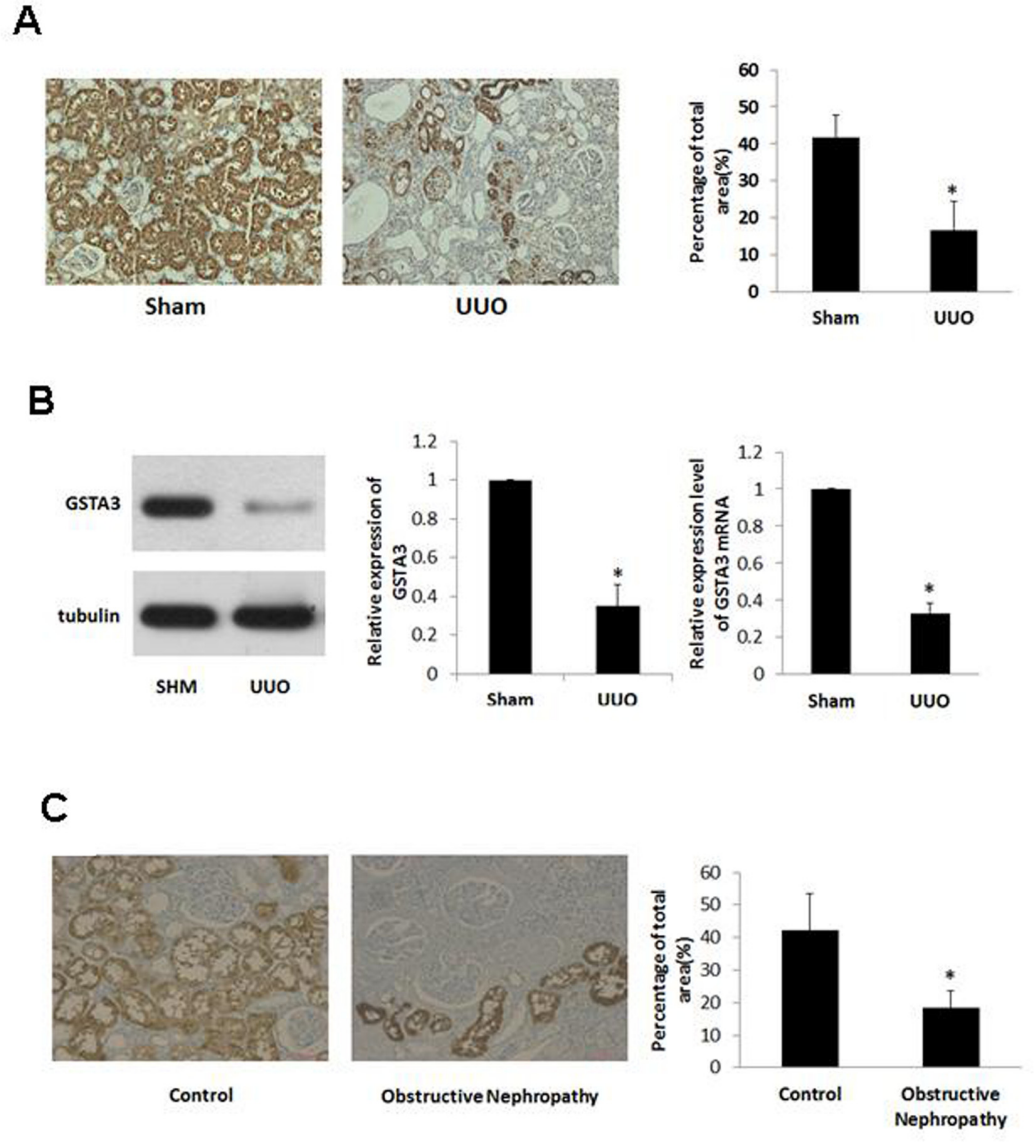
**A) Representative images of the cellular localization of GSTA3 in renal cortex of rats (×200), and quantitative analysis of GSTA3 expression.** B) The expression of GSTA3 protein and mRNA. C) The cellular localization of GSTA3 in renal cortex of human (×200), and quantitative analysis of GSTA3 expression. Data are expressed as means±S.E of three independent experiments. *p<0.05 vs. Sham.

To assess the potential target proteins involved in RIF, we extracted renal cortex protein of UUO rats for proteomics analysis. By using high-throughput iTRAQ screening, 1105 proteins were identified. United Affymetrix GeneChip profiling revealed that 27 protein expressions were significantly reduced (fold-change<0.5) in the renal cortex of UUO rats compared to the corresponding controls (Table 2). GSTA3 was the most significantly down-regulated one among all differentially expressed proteins. GSTA3 expression level in UUO rats was significantly reduced comparing to SHM rats (Fig 2A). We have further validated the result using Western blot and Real Time-PCR methods. The result suggested that GSTA3 protein and mRNA expression levels were significantly decreased in renal cortex of UUO rats (Fig 2B).

**Table 2:** Differentially expressed proteins in proteomics analysis.

	Gene Symbol	Protein Name	Swiss-prot accession number	UUO/S HM
1	Pc	Pyruvate carboxylase, mitochondrial	P52873	0.497
2	Prdx1	Peroxiredoxin-1	Q63716	0.492
3	Ddah1	N(G),N(G)-dimethylarginine dimethylaminohydrolase 1	O08557	0.483
4	Atp6v1f	V-type proton ATPase subunit F	P50408	0.461
5	Pgam1	Phosphoglycerate mutase 1	P25113	0.453
6	Ezr	Ezrin	P31977	0.445
7	Plvap	Plasmalemma vesicle-associated protein	Q9WV78	0.437
8	Dhtkd1	Probable 2-oxoglutarate dehydrogenase E1 component DHKTD1, mitochondrial	Q4KLP0	0.409
9	Got2	Aspartate aminotransferase, mitochondrial	P00507	0.406
10	Hadhb	Trifunctional enzyme subunit beta mitochondrial	Q60587	0.391
11	Echs1	Enoyl-CoA hydratase, mitochondrial	P14604	0.391
12	Mdh1	Malate dehydrogenase, cytoplasmic	O88989	0.38
13	Gsta1	Glutathione S-transferase alpha-1	P00502	0.377
14	Pebp1	Phosphatidylethanolamine-binding protein 1	P31044	0.377
15	Dab2	Disabled homolog 2	O88797	0.373
16	Atp5h	ATP synthase subunit d, mitochondrial	P31399	0.37
17	Sptan1	Spectrin alpha chain, brain	P16086	0.331
18	Fbp1	Fructose-1,6-bisphosphatase 1	P19112	0.311
19	Sod1	Superoxide dismutase [Cu-Zn]	P07632	0.288
20	Hrsp12	Ribonuclease UK114	P52759	0.286
21	Hao2	Hydroxyacid oxidase 2	Q07523	0.283
22	Hao2	Hydroxyacid oxidase 2	Q07523	0.283
23	Etfa	Electron transfer flavoprotein subunit alpha mitochondrial	P13803	0.242
24	Atp5j	ATP synthase-coupling factor 6, mitochondrial	P21571	0.211
25	Glyctk	Glycerate kinase	Q0VGK3	0.2
26	Hspd1	60 kDa heat shock protein	P63039	0.188
27	Gsta3	Glutathione S-transferase alpha-3	P04904	0.182

UUO/SHM: Ratio of UUO contrast to SHM rats

Till now, we have collected renal tissues from eight normal and eight obstructive nephropathy kidneys and measured GSTA3 protein expressions using immunohistochemical analysis. We have found that GSTA3 protein expression was significantly reduced in obstructive nephropathy kidneys (*p*<0.05) (Fig 2C)

### TGF-beta-induced tubular EMT was correlated with GSTA3

**Fig 3:**
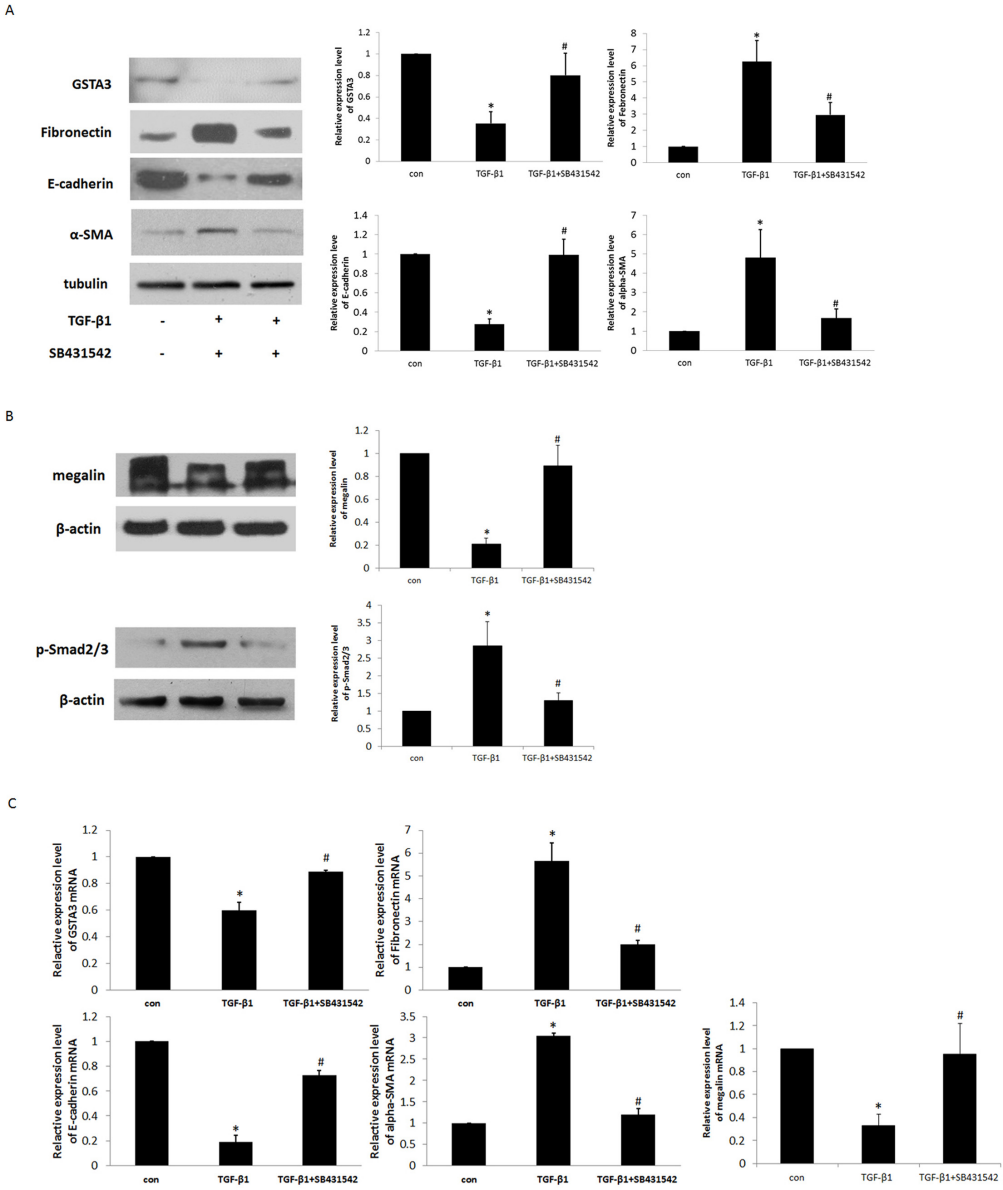
**A) Effects of TGF-β1 on GSTA3, FN, α-SMA, E-cadherin, megalin and p-Smad2/3 protein expressions in NRK-52E cells.** Serum-starved NRK-52E cells were stimulated with 10 ng/ml TGF-β1 for 48 h. SB431542 (10ng/ml) was administered 1 h prior to the addition of TGF-β1. The expression of GSTA3, FN, α-SMA, and E-cadherin was subjected to western blot analysis. **B) Effects of TGF-β1 on GSTA3, FN, α-SMA, E-cadherin and megalin mRNA expressions in NRK-52E cells.** The mRNA from NRK-52E cells was extracted, and the mRNA expression for GSTA3, α-SMA, and E-cadherin was detected by real-time PCR.

TGF-beta, a fibrosis stimulator, is able to induce EMT in cultured epithelial cells [23]. Although GSTA3 and E-cadherin were highly expressed in NRK-52E cells, there was no detectable expression of α-SMA and FN in these cells. TGF-β1 treatment down-regulated GSTA3, E-cadherin and megalin protein and mRNA expression but up-regulated α-SMA, FN and p-Smad2/3 protein and mRNA expression in NRK-52E cells, with a peak at 10 ng/ml at day 2 (Fig 3A and 3B). These were confirmed using TGF-β1 superfamily type I activin receptor-like kinase inhibitor (SB431542), which significantly inhibits the actions of TGF-β1. Pre-treatment with SB431542 resulted in the increase in GSTA3, E-cadherin and megalin protein and mRNA expressions, and the attenuation of α-SMA, FN and p-Smad2/3 protein and mRNA expression (Fig 3A and 3B).

### Protection against TGF-β1-induced Renal Tubular EMT by GSTA3

**Fig 4:**
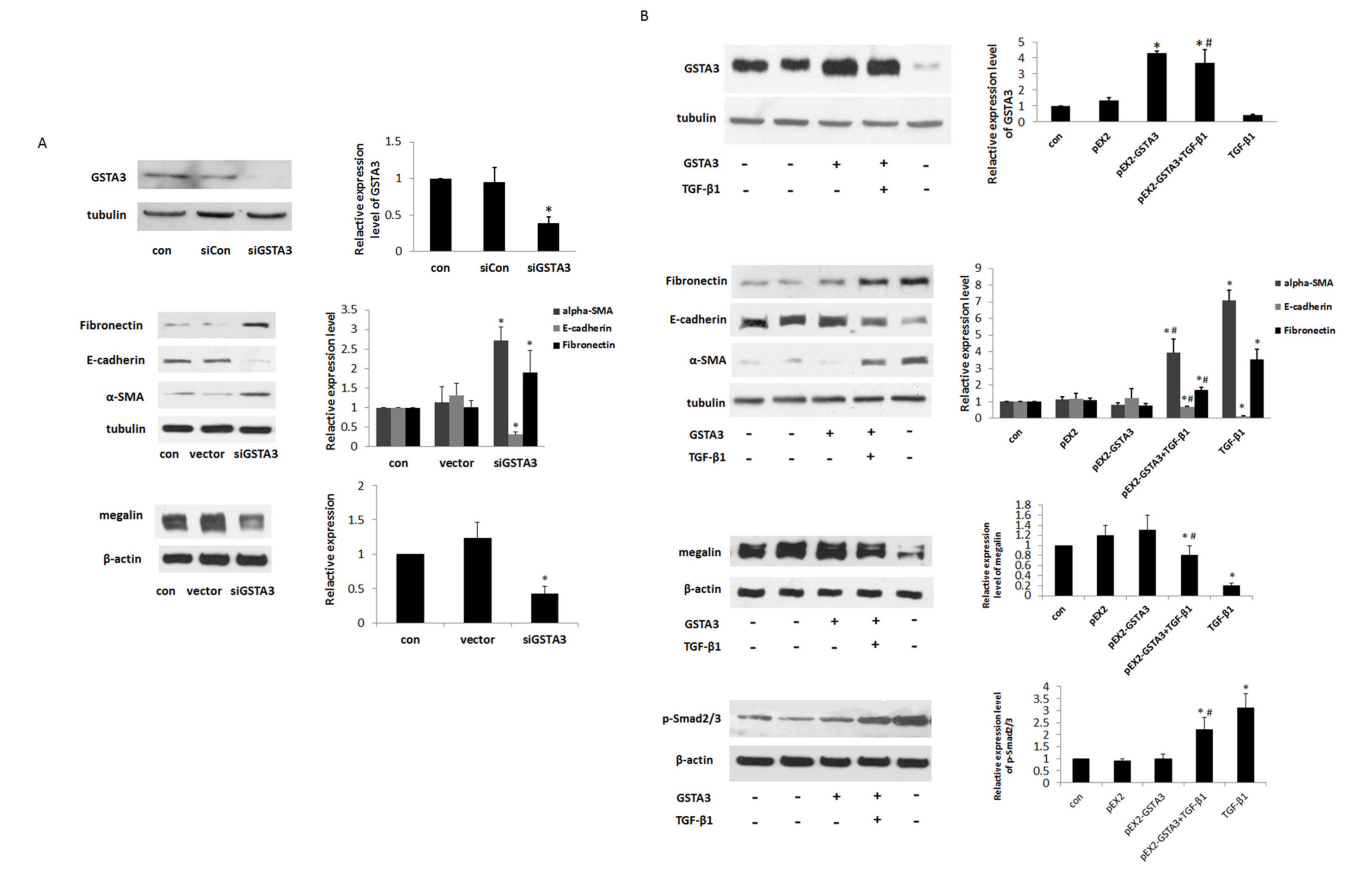
**A) Effects of knocking down GSTA3 on EMT in NRK-52E cells.** After a 6 h incubation of cells with siRNA-Lipofectamine 2000 complex, the cells were maintained with normal DMEM for an additional 18 h. Cells were treated with TGF-β1 and harvested for GSTA3, FN, E-cadherin, α-SMA and megalin protein expression after 48 h. **B) Effects of modulation of GSTA3 over-expression on EMT in TGF-β1 treated NRK-52E cells.** After the transfected cells were treated with 10 ng/ml TGF-β1 for 48 h, GSTA3, FN, E-cadherin, α-SMA, megalin and p-Smad2/3 protein expression were analyzed by western blot. Results are presented as the means±S.E. of three independent experiments. *p<0.05 vs. control; #p<0.05 vs. TGF-β1.

To further understand the role of GSTA3 in tubular EMT, GSTA3 gene was knocked down by GSTA3 siRNA transfection. The cells transfected with GSTA3 siRNA exhibited greater sensitivity towards EMT compared to those transfected with control siRNA (Fig 4A). The cells transfected with GSTA3 siRNA showed significant increases in α-SMA (*p*<0.05), whereas E-cadherin and megalin expressions were significantly decreased (*p*<0.05) A significant increase in FN was also observed in the GSTA3 siRNA-transfected cells.

The cells were transfected with pEX2-GSTA3 to explore whether over-expression of GSTA3 could suppress TGF-β1-induced renal tubular EMT (Fig 4B). The proteins were harvested for Western blot analysis after treatment with TGF-β1 for 48 h. Transient transfection with pEX2-GSTA3 plasmid increased the cytoplasmic GSTA3 levels compared to control (*p*<0.05). Transfecting cells with pEX2-GTAS3 resulted in the down-regulation of α-SMA, FNs but up-regulation of E-Cadherin and megalin expression, which suggests a protective role of GSTA3 against TGF-β1-induced EMT.

### GSTA3 protection against TGF-β1-induced oxidative damage in NRK-52E cells

**Fig 5:**
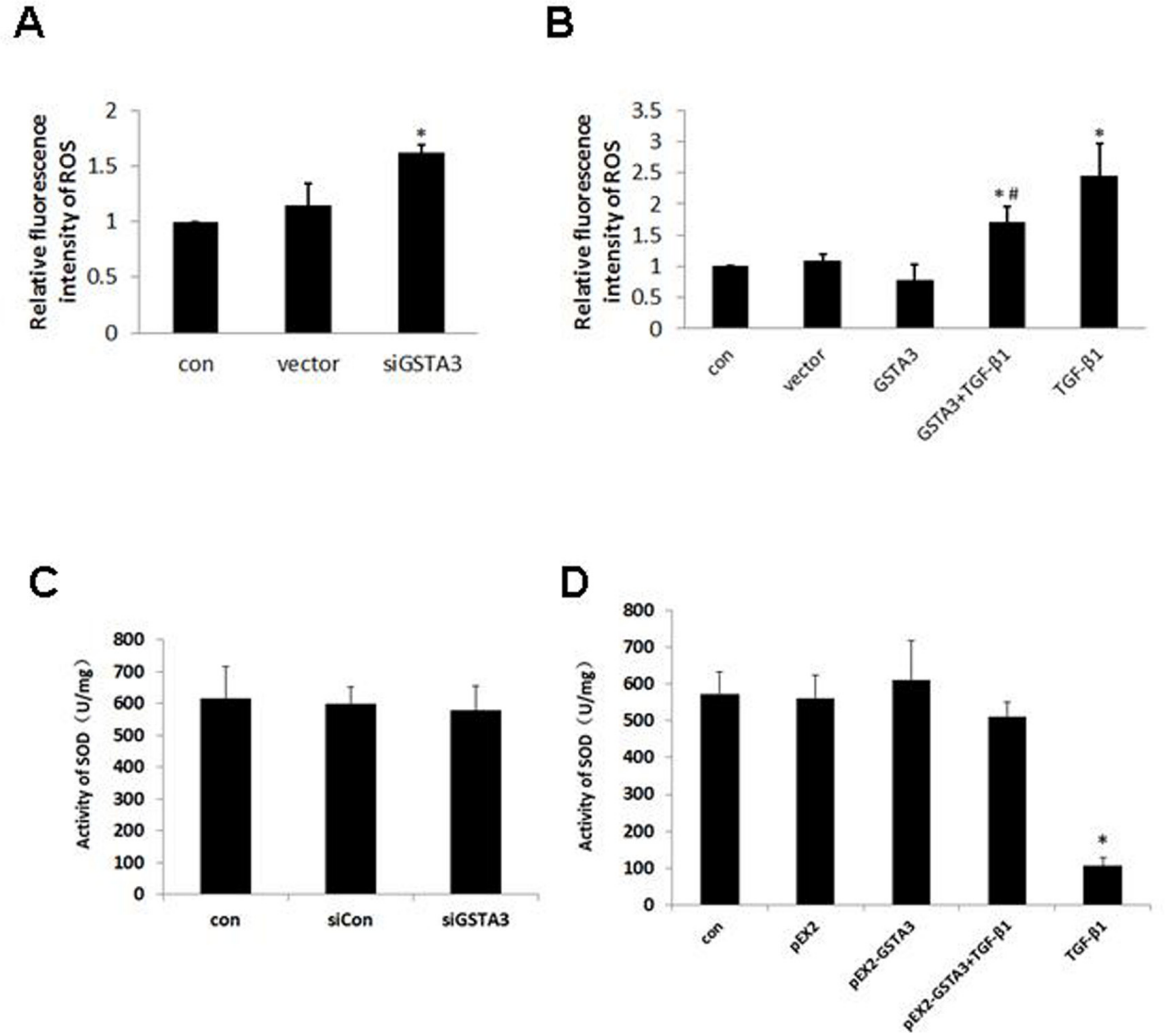
Effect of GSTA3 on NRK-52E cellular ROS. A) ROS level of knocking down GSTA3 in NRK-52E; B) ROS level of treatment with 10 ng/ml TGF-β1 for 15min in GSTA3 over-expression cells. C) SOD activity knocking down GSTA3 in NRK-52E; D) SOD activity of treatment with 10 ng/ml TGF-β1 for 15min in GSTA3 over-expression cells. Results are presented as the means±S.E. of 3 independent experiments. *p<0.05 vs. control; ^#^p<0.05 vs. TGF-β1.

To determine whether GSTA3-regulated ROS production and SOD activity is associated with TGF-β1-induced oxidative damage, the change of ROS production and SOD activity was determined following GSTA3 overexpression and GSTA3 gene silencing. As expected, ROS production was significantly increased, while SOD activity unchanged in GSTA3 siRNA-transfected cells (*p*<0.05) (Fig 5A and 5C). Treating cells with 10 ng/ml TGF-β1 for 15 min resulted in an increase in ROS production and a decrease in SOD activity in NRK-52E cells (*p*<0.05) (Fig 5B and 5D). In contrast, the ROS levels were significantly decreased by GSTA3 overexpression prior to the TGF-β1 treatment (*p*<0.05). These results suggested that inhibition of ROS production is associated with the protective role of GSTA3 against TGF-β1-induced EMT.

## Discussion

RIF is the common pathway by which all the various CKD progress leading to end-stage renal disease (ESRD). The severity of renal tubular interstitial injury determines the prognosis of CKD. RIF is a pathological process inducing by a variety of factors, included oxidation stress, inflammation, fibroblasts proliferation and activation, EMT, cytokines and apoptosis. Although the exact role of EMT in renal fibrosis remains to be established [24], it is widely believed that renal tubular EMT is one of the critical factors mediating RIF. Oxidative stress damage is associated with the pathogenesis of RIF by tubular EMT [25].

The molecular mechanisms driving fibrogenesis are not fully understood. Through the proteomics analysis, we found that the introduction of RIF was accompanied by significant reduction in GSTA3. Thus far, the six classes of glutathione S-transferases (GSTs) have been described, Alpha, Mu, Pi, Sigma, Theta and Zeta. The rat GSTA3 (rGST Yc1) subunit, a member of glutathione S-transferases (GSTs) family, belongs to the Alpha class, and is located on chromosome 9 [26]. It widely exists in animals, plants, microorganisms, and other biological tissues. It is mainly distributed in liver, kidney, small intestine, gastrointestinal tract, and other organs [27]. It was reported that extensive liver fibrosis are observed in GSTA3 knockout mice [12], suggesting that GSTA3 may be critical for protecting mice from liver fibrosis. However, the link between GSTA3 and RIF remained to be determined.

The UUO operation used in this study provides a rodent model of fibrotic nephropathy resembling human CKD. In fibrotic UUO kidneys, the interstitial spaces filled with fibrillar materials consisting of collagens type I and FN. We found that GSTA3 protein and mRNA expression was decreased in UUO rats. TGF-β is believed to be an essential fibrogenic factor. *In vitro*, inhibiting TGF-β1R signaling potentiates GSTA3 expression, suggesting that GSTA3 plays a role in RIF through TGF-β1 pathway. To further confirm it, we knocked down and overexpression GSTA3 in NRK-52E cells. We found knocking down GSTA3 exacerbated EMT, whereas GSTA3 overexpression attenuated TGF-β1 induced RIF, all to suggest that GSTA3 prevent RIF by reducing EMT. These *in vitro* findings are consistent with the induction of RIF companied with decreased GSTA3 expression *in vivo* by UUO operation.

Oxidative stress is one of the important pathogenesis of RIF in which EMT plays a crucial role. The *in vitro* work of the present study showed decreases in superoxide dismutase (SOD) activity increases renal tissue collagen deposition, α-SMA expression, and decreases in E-Cadherin and RIF in UUO rats. ROS can directly affect renal tubular epithelial cells by inducing cellular shape changes, α-SMA expression and E-Cadherin loss [11]. It was shown that hepatic GST expression may be decreased during diabetes, and that GSTs may protect mice from diabetes and its complications through the mechanisms of anti-oxidative stress [28]. GSTA3 gene expression was significantly increased by antioxidant therapy in H4IIE cells [29]. GSTA3 protein expression was also increased by the broccoli seed extract in Nrf2^+/+^ mouse embryonic fibroblasts [30]. We found oxidation stress was enhanced in GSTA3 knock down NRK-52E cells, and ROS activity was reduced in GSTA3 overexpression cells. Taken together, these findings suggest significant interrelationships between GSTA3 expression and oxidative stress in renal disease.

In summary, the findings of the present study indicate that GSTA3 is a major regulator for RIF which may be mediated by oxidative stress-induced EMT and that overexpression of GSTA3 may be one of the mechanisms by which a blockade of the EMT process confers renoprotective effects.

## References

[1] Boor P, Ostendorf T, Floege J. Renal fibrosis: Novel insights into mechanisms and therapeutic targets. Nat. Rev. Nephrol. 2010;6:643–56. 10.1038/nrneph.2010.120 20838416

[2] Kalluri R, Weinberg RA. The basics of epithelial-mesenchymal transition. J Clin Invest. 2009;119:1420–28. 10.1172/JCI39104 19487818 PMC2689101

[3] Radisky DC, Kenny PA, Bissell MJ. Fibrosis and cancer: do myofibroblasts come also from epithelial cells via TUBULAR EMT? J Cell Biochem. 2007;101:830–39. 17211838 10.1002/jcb.21186 PMC2838476

[4] Li Q, Lv LL, Wu M, Zhang XL, Liu BC, Liu H. Dexamethasone prevents monocyte-induced tubular epithelial-mesenchymal transition in HK-2 cells. J Cell Biochem. 2012;11:415–23. 10.1002/jcb.24405 23060286

[5] Djamali A, Reese S, Yracheta J, Oberley T, Hullett D, Becker B. Epithelial-to-mesenchymal transition and oxidative stress in chronic allograft nephropathy. Am J Transplant. 2005;5:500–9. 15707404 10.1111/j.1600-6143.2004.00713.x

[6] Liu Y. Renal fibrosis: new insights into the pathogenesis and therapeutics. Kidney Int. 2006;69:213–17. 16408108 10.1038/sj.ki.5000054

[7] Djamali A. Oxidative stress as a common pathway to chronic tubulointerstitial injury in kidney allografts. Am J Physiol Renal Physiol. 2007;293:F445–55. 17459952 10.1152/ajprenal.00037.2007

[8] Liu Q, Liu S, Shi Y, Li H, Hao J, Xing L, et al Suppressors of cytokine signaling inhibit tubular epithelial cell myofibroblast transdifferentiation. Am J Nephrol. 2011;34:142–51. 10.1159/000329325 21734367

[9] Nlandu Khodo S, Dizin E, Sossauer G, Szanto I, Martin PY, Feraille E, et al NADPH-Oxidase 4 Protects against Kidney Fibrosis during Chronic Renal Injury. J Am Soc Nephrol. 2012;23:1967–76. 10.1681/ASN.2012040373 23100220 PMC3507365

[10] Vaziri ND, Lin CY, Farmand F, Sindhu RK. Superoxide dismutase, catalase, glutathione peroxidase and NADPH oxidase in lead-induced hypertension. Kidney International. 2003;63:186–94. 12472782 10.1046/j.1523-1755.2003.00711.x

[11] Rhyu DY, Yang Y, Ha H, Lee GT, Song JS, Uh ST, et al Role of reactive oxygen species in TGF-beta1-induced mitogen-activated protein kinase activation and epithelial-mesenchymal transition in renal tubular epithelial cells. J Am Soc Nephrol. 2005;16(3):667–75. 15677311 10.1681/ASN.2004050425

[12] Ilic Z, Crawford D, Egner PA, Sell S. Glutathione-S-transferase A3 knockout mice are sensitive to acute cytotoxic and genotoxic effects of aflatoxin B1. Toxicology & Applied Pharmacology. 2010;242(3):241–46. 19850059 10.1016/j.taap.2009.10.008 PMC2813954

[13] Gonzalez J, Mouttalib S, Delage C, Calise D, Maoret JJ, Pradère JP, et al Dual effect of chemokine CCL7/MCP-3 in the development of renal tubulointerstitial fibrosis. Biochem Biophys Res Commun. 2013;23;438(2):257–63. 10.1016/j.bbrc.2013.07.025 23872063 PMC4043121

[14] Lan HY, Mu W, Tomita N, Huang XR, Li JH, Zhu HJ, et al Inhibition of renal fibrosis by gene transfer of inducible Smad7 using ultrasound microbubble system in rat UUO model. J Am Soc Nephrol. 2003;14:1535–48. 12761254 10.1097/01.asn.0000067632.04658.b8

[15] Unwin Richard D, Griffiths John R, Whetton Anthony D. Simultaneous analysis of relative protein expression levels across multiple samples using iTRAQ isobaric tags with 2D nano LC–MS/MS. Nature Protocols. 2010;5:1574–82. 10.1038/nprot.2010.123 21085123

[16] Radford MG Jr, Donadio JV Jr, Bergstralh EJ, Grande JP. Predicting renal outcome in IgA nephropathy. J Am Soc Nephrol. 1997;8:199–207. 9048338 10.1681/ASN.V82199

[17] Lin SL, Chen RH, Chen YM, Chiang WC, Lai CF, Wu KD, et al Pentoxifylline attenuates tubulointerstitial fibrosis by blocking Smad3/4-activated transcription and profibrogenic effects of connective tissue growth factor. J Am Soc Nephrol. 2005;16:2702–13. 15987746 10.1681/ASN.2005040435

[18] Broekema M, Harmsen MC, van Luyn MJ, Koerts JA, Petersen AH, van Kooten TG, et al Bone marrowderived myofibroblasts contribute to the renal interstitial myofibroblast population and produce procollagen I after schemia/reperfusion in rats. J Am Soc Nephrol. 2007;18:165–75. 17135399 10.1681/ASN.2005070730

[19] Hwang M, Kim HJ, Noh HJ, Chang YC, Chae YM, Kim KH, et al TGF-beta1 siRNA suppresses the tubulointerstitial fibrosis in the kidney of ureteral obstruction. Exp Mol Pathol. 2006;81:48–54. 16443218 10.1016/j.yexmp.2005.11.005

[20] Zhang X, Li Y, Dai C, Yang J, Mundel P, Liu Y. Sp1 and Sp3 transcription factors synergistically regulate HGF receptor gene expression in kidney. Am J Physiol Renal Physiol. 2003;284:F82–F94. 12473536 10.1152/ajprenal.00200.2002

[21] Zeisberg M, Neilson EG. Biomarkers for epithelial-mesenchymal transitions. J Clin Invest. 2009;119(6):1429–37. 10.1172/JCI36183 19487819 PMC2689132

[22] Grande MT, López-Novoa JM. Fibroblast activation and myofibroblast generation in obstructive nephropathy. Nature Reviews Nephrology. 2009;5:319–28. 10.1038/nrneph.2009.74 19474827

[23] Bottinger EP, Bitzer M. TGF-β signaling in renal disease. J Am Soc Nephrol. 2002;13:2600–10. 12239251 10.1097/01.asn.0000033611.79556.ae

[24] Humphreys BD, Lin SL, Kobayashi A, Hudson TE, Nowlin BT, Bonventre JV, et al Fate tracing reveals the pericyte and not epithelial origin of myofibroblasts in kidney fibrosis. Am J Pathol. 2010;176(1):85–97. 10.2353/ajpath.2010.090517 20008127 PMC2797872

[25] Zhang X, Liang D, Chi ZH, Chu Q, Zhao C, Ma RZ, et al Effect of zinc on high glucose-induced epithelial-to-mesenchymal transition in renal tubular epithelial cells. Int J Mol Med. 2015;35(6):1747–54. 10.3892/ijmm.2015.2170 25872526

[26] Yamada T, Muramatsu Y, Yasue M, Agui T, Yamada J, Matsumoto K. Chromosomal assignments of genes for rat glutathione S-transferase Ya (GSTA1) and Yc subunits (GSTA2). Cytogenet Cell Genet. 1992;61(2):125–27. 1395720 10.1159/000133388

[27] Fotouhi-Ardakani N, Batist G. Genomic cloning and characterization of the rat glutathione S-transferase -A3-subunit gene. Biochem J. 1999;339:685–93. 10215608 PMC1220205

[28] Kim SK, Woodcroft KJ, Novak RF. Insulin and glucagon regulation of glutathione S-transferase expression in primary cultured rat hepatocytes. J Pharmacol Exp Ther. 2003;305(1):353–61. 12649389 10.1124/jpet.102.045153

[29] Gum SI, Jo SJ, Ahn SH, Kim SG, Kim JT, Shin HM, et al The potent protective effect of wild ginseng (Panax ginseng C.A. Meyer) against benzo[alpha]pyrene-induced toxicity through metabolic regulation of CYP1A1 and GSTs. J Ethnopharmacol. 2007;112(3):568–76. 17590295 10.1016/j.jep.2007.05.014

[30] McWalter GK, Higgins LG, McLellan LI, Henderson CJ, Song L, Thornalley PJ, et al Transcription factor Nrf2 is essential for induction of NAD(P)H: quinone oxidoreductase 1, glutathione S-transferases, and glutamate cysteine ligase by broccoli seeds and isothiocyanates. J Nutr. 2004;134(12):3499S–506S. 15570060 10.1093/jn/134.12.3499S

